# Current state and practice variation in the use of Meningitis/Encephalitis (ME) FilmArray panel in children

**DOI:** 10.1186/s12879-022-07789-2

**Published:** 2022-10-31

**Authors:** Prabi Rajbhandari, Nathaniel Goodrich, Aleisha M. Nabower, Miraides F. Brown, Maheswari Ekambaram, Jaclyn Eisenberg, Michael L. Forbes, Nathan Gollehon, Kimberly C. Martin, Russell McCulloh, Bryan Stone, Matt Tandy, Jessica Snowden

**Affiliations:** 1 Akron Children’s Hospital, One Perkins Square, Akron, OH USA; 2 University of Nebraska Medical Center, Omaha, NE USA; 3 Baylor Scott &White Medical Center, 300 University Blvd, Round Rock, TX USA; 4 The University of Chicago Medicine, 5841 S. Maryland Avenue, Chicago, IL USA; 5 University of Oklahoma-Tulsa School of Community Medicine, Tulsa, OK USA; 6 University of Utah Health, 50 North Medical Drive, Salt Lake City, UT USA; 7 University of Arkansas for Medical Sciences Little Rock, Arkansas, USA

## Abstract

**Background:**

The Meningitis/Encephalitis FilmArray® Panel (ME panel) was approved by the U.S. Food and Drug Administration in 2015 and provides rapid results when assessing patients with suspected meningitis or encephalitis. These patients are evaluated by various subspecialties including pediatric hospital medicine (PHM), pediatric emergency medicine (PEM), pediatric infectious diseases, and pediatric intensive care unit (PICU) physicians. The objective of this study was to evaluate the current use of the ME panel and describe the provider and subspecialty practice variation.

**Methods:**

We conducted an online cross-sectional survey via the American Academy of Pediatrics Section of Hospital Medicine (AAP-SOHM) ListServe, Brown University PEM ListServe, and PICU Virtual pediatric system (VPS) Listserve.

**Results:**

A total of 335 participants out of an estimated 6998 ListServe subscribers responded to the survey. 68% reported currently using the ME panel at their institutions. Among test users, most reported not having institutional guidelines on test indications (75%) or interpretation (76%). 58% of providers self-reported lack of knowledge of the test’s performance characteristics. Providers from institutions that have established guidelines reported higher knowledge compared to those that did not (51% vs. 38%; p = 0.01). More PHM providers reported awareness of ME panel performance characteristics compared to PEM physicians (48% vs. 27%; p = 0.004); confidence in test interpretation was similar between both groups (72 vs. 69%; p = 0.80).

**Conclusion:**

Despite the widespread use of the ME panel, few providers report having institutional guidelines on test indications or interpretation. There is an opportunity to provide knowledge and guidance about the ME panel among various pediatric subspecialties.

**Supplementary Information:**

The online version contains supplementary material available at 10.1186/s12879-022-07789-2.

## Introduction

Pediatric meningitis and encephalitis (ME) are serious illnesses with significant morbidity and mortality including long-term sequelae with considerable economic burden [1, 2]. In October 2015, the United States (U.S.) Food and Drug Administration approved the Meningitis/Encephalitis FilmArray® Panel (ME panel), a test that offers rapid results compared to cerebrospinal fluid (CSF) culture for evaluation of suspected pediatric meningitis or encephalitis [3]. The test was developed by BioFire® [4] Diagnostics (Salt Lake City, Utah) and is a qualitative multiplex polymerase chain reaction (PCR) test used on the Film Array system. The ME panel detects 14 pathogens in the CSF: *Escherichia coli* K1, *Haemophilus influenzae, Listeria monocytogenes, Neisseria meningitidis, Streptococcus agalactiae* (group B streptococcus or GBS), *Streptococcus pneumoniae*, Cytomegalovirus (CMV), Enterovirus, Epstein-Barr virus (EBV), Herpes simplex virus types 1 and 2 (HSV1 and HSV2), Human Herpes virus 6 (HHV6), Varicella zoster virus (VZV), Human parechovirus, and *Cryptococcus neoformans/gatti*. ME panel sensitivities vary by molecular target, with pediatric studies demonstrating sensitivities of 84–96% and specificities of > 98% for the ME panel relative to the gold standard CSF cultures or standalone PCR testing [5–7]. The ME panel is also regarded as a more sensitive test than culture if the patient has been pre-treated with antibiotics prior to CSF collection [7, 8].

Clinical management of suspected meningitis/encephalitis depends on the specific pathogens causing illness and ranges from supportive care to a prolonged intensive care unit (ICU) stay and antimicrobial therapy [9–11]. While a timely diagnosis of central nervous system (CNS) infections is critical, ruling out infection can impact patient care, including reducing healthcare cost, length of stay, extended and unnecessary antimicrobial therapy, and procedures [5, 12].

Currently, there are no published guidelines regarding indications for the use of the ME panel or interpretation of results. Patients with suspected meningitis and encephalitis are cared for by various subspecialties, including but not limited to, Pediatric Hospital Medicine (PHM), Pediatric Emergency Medicine (PEM), Pediatric Infectious Diseases (ID), and Pediatric Intensive Care Unit (PICU) physicians through the course of illness. Little is known about the current state of use of the panel in the U.S and the practice variation among institutions and sub-specialties. Practice variation occurs when there is uncertainty in diagnosis/management, and no clear evidence or guidelines exist. Recognizing that variation exists is the first step in standardizing the best practice of how the ME panel should be used.

The purpose of this study was to evaluate the current use of ME panel and to explore provider and subspecialty practice variation in its use.

## Methods

The study was approved by the Institutional Review Board at the Akron Children’s Hospital, Akron, Ohio, USA. We performed an online cross-sectional survey of PHM, PEM, and PICU physicians between August-September of 2019. The survey was created by an iterative process among physicians from the above-mentioned specialties with additional input from ID physicians. The survey was pilot tested among 50 PHM providers and adjustments made to improve the questions’ clarity. The final survey consisted of 14 items, including demographic information and two specific clinical scenarios (supplemental file) and was created on Institutional Research Electronic Data Capture (REDCap). The survey was distributed through the American Academy of Pediatrics Section on Hospital Medicine (AAP-SOHM) (4074 subscribers), Brown University PEM (2724 subscribers), and PICU virtual pediatric system (VPS) (200 PICU’s with one contact person at each site) listservs. The AAP-SOHM listserve is sponsored by the AAP and is targeted to pediatric hospitalists. The Brown University PEM listserve is sponsored by Brown University, but targets PEM providers around the country. The PICU VPS listserve represents 200 PICU’s. In total, we estimate that there are 6998 subscribers to these listserves. However, there are frequent changes to subscribers and, therefore, total numbers are hard to definitively identify. Providers subscribed to these listserves above received an email with a unique online survey from REDCap. Reminder emails were sent out twice, 2-weeks apart. An opportunity to enter a raffle to win one of four $25 gift cards was provided to increase survey response rates.

Statistical analysis was performed using SAS (version 9.4; SAS Institute Inc., Cary, NC, USA). Descriptive statistics were used as appropriate. Chi-square test and/or Fisher’s exact tests were used to compare categorical variables. All tests were two-sided, and P-value less than 0.05 was considered significant.

## Results

### Participants characteristics

**Fig. 1:**
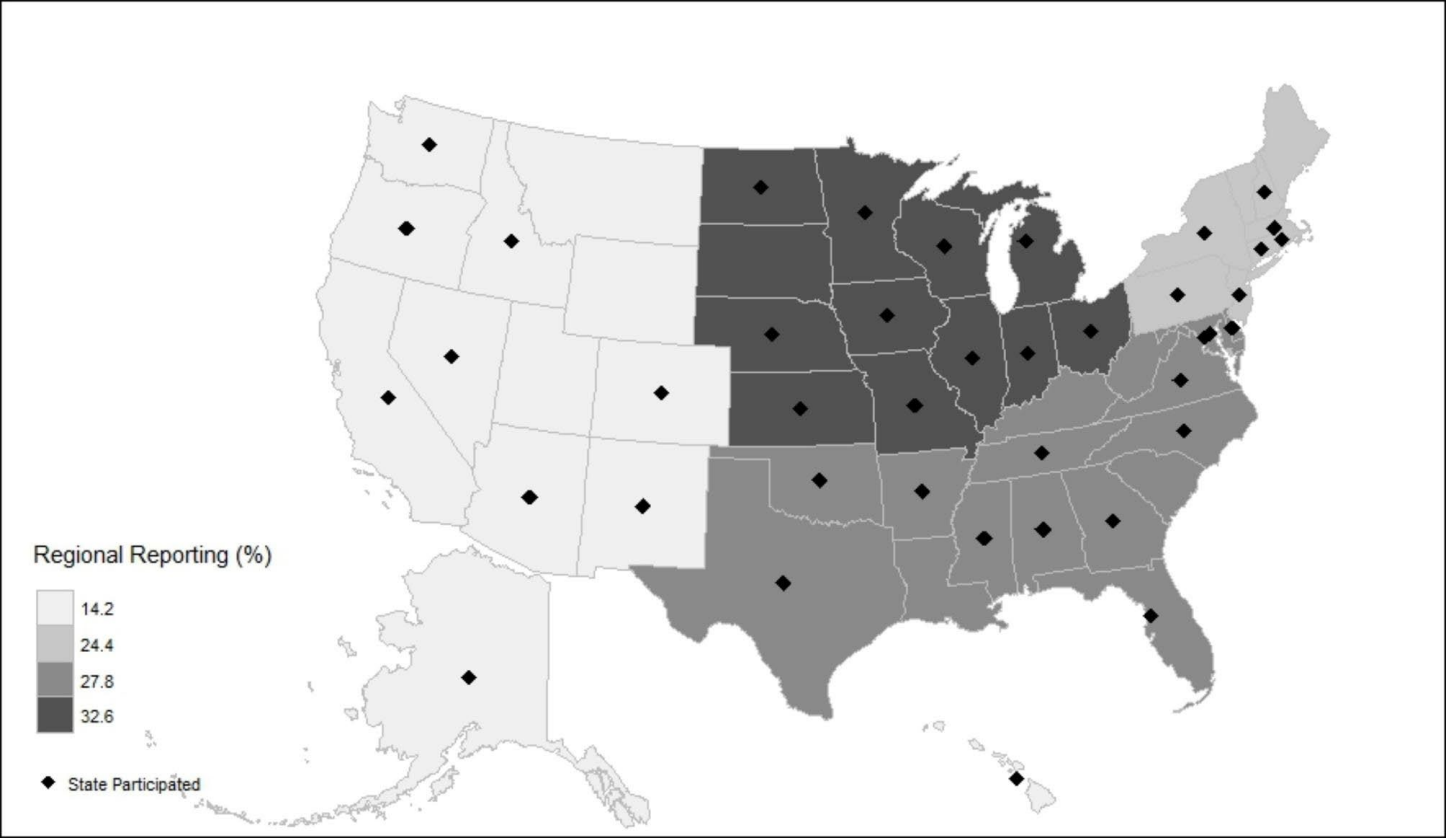
Figure showing various participating states in USA, states with diamond sign ◆ shows at least 1 response from corresponding state

A total of 335 providers (4.8% response rate) from 40 states and 177 hospitals responded to the survey (Fig. 1). Four providers did not complete the entire survey and were excluded from the final count of 331 responses. Sixty-five providers did not identify their hospital on the survey. There were 169 PHM, 130 PEM, 19 PICU, 4 ID, and 1 NICU providers who responded to this survey. The majority of respondents were PHM providers (n = 169, 51%), worked in a university-affiliated children’s hospital (n = 193, 58%), and 39% (n = 56) were in practice for more than 10 years (Table 1). Of the two-hundred twenty-six providers who used the ME panel, 58% self-reported a lack of knowledge of performance characteristic of the ME panel. In terms of ability to interpret the test results, 71% (159/226) self-reported being confident or very confident in interpreting the test result.

**Table 1:** Demographics of the survey respondents

		N	%
Characteristics of hospital			
	Children’s hospital - Not university affiliated	46	13.9
	Children’s hospital - University affiliated	193	58.3
	Community hospital - Not university hospital	36	10.8
	Community hospital - University affiliated	41	12.3
	Others	15	4.53
Location			
	Outside United states	3	0.91
	US-Midwest	108	32.6
	US-Northeast	81	24.4
	US-West coast	47	14.2
	US-South	92	27.7
Your current position			
	Physician (MD/DO)	320	97.5
	Advance practice provider	8	2.4
	No answer	3	
Years in practice			
	In-Training	20	6.0
	1–2 years	51	15.4
	3–5 years	73	22.1
	6–10 years	56	16.9
	10 + years No answer	130 1	39.3
Sub-specialty			
	PHM	169	51.2
	PEM	130	39.3
	PICU	19	5.7
	ID	4	1.2
	NICU	1	0.3
	Others	8	2.1

Abbreviations: PHM, pediatric hospital medicine; PEM, pediatric emergency medicine; PICU, pediatric critical care unit ;ID, infectious disease; NICU, neonatal intensive care unit

### Current use of ME panel

Most participants (68%, 226/331) reported using the ME panel at their institution with fewer (8%, 28/331) using the test for more than four years. Among those who use the ME panel, 4% reported still using a single organism PCR test in all scenarios, 55% reported using it in some scenarios and 40% reported that they no longer use a single organism PCR test. 52% (116/226) reported that the result was available in < 3 h, with most (78%) reporting that results were available within 6 h. A prolonged test result time was statistically associated with the test being performed as a send-out from the institution (p = 0.001).

### Availability of guidelines

Among those using the panel, 75% (171/226) and 76% (173/226) of respondents reported not having institutional guidelines about utilization and interpretation of ME panel results, respectively (Table 2). There was no difference noted between free-standing children’s hospitals and community hospitals in terms of availability of guidelines (24% vs. 21%; p = 0.62). Similarly, no difference was noted with university affiliation in terms of providing guidelines for ordering and interpreting results (17% vs. 26%; p = 0.15). A subset analysis was done between providers from institutions that provided guidelines and those that did not. Providers from institutions that provided guidelines self-reported higher knowledge of test performance characteristics as compared to those that did not (51% vs. 38%; p = 0.01). There was no difference in reported confidence in interpreting ME panel results between these two groups (p = 0.09). No difference was noted in terms of starting, stopping, or narrowing antibiotics (p = 0.22; p = 0.36; p = 0.70), respectively, between these two groups.

**Table 2:** Responses to institutional and personal awareness/confidence questions, in addition to responses to clinical case scenarios

	Total N (%)	Specialty N (%)			
		Hospitalist	ED	Others	*P*
Are you aware of any institutional guidelines regarding the use (ordering) of CSF BioFire® testing?	-	-	-	-	0.2585
Yes	55 (24.3)	35 (27.3)	14 (18.0)	6 (30.0)	
No	171 (75.7)	93 (72.7)	64 (82.0)	14 (70.0)	
Does your institution provide guidelines for interpreting CSF BioFire® results?	-	-	-	-	0.582
Yes	53 (23.5)	27 (21.1)	20 (25.60	6 (30.0)	
No	173 (76.5)	101 (78.9)	58 (74.4)	14 (70.0)	
Are you aware of the performance characteristics (sensitivity/specificity/positive predictive value/negative predictive value) of the CSF BioFire® test?	-	-	-	-	**0.0366**
Yes	36 (16.0)	23 (18.0)	8 (10.4)	5 (25.0)	
No	132 (58.7)	67 (52.3)	56 (72.7)	9 (45.0)	
Depends on organism	57 (25.3)	38 (29.7)	13 (16.9)	6 (30.0)	
On a scale of 1 to 5, with 5 being VERY confident, how confident are you in interpreting the results of the CSF BioFire® test?	-	-	-	-	0.9394
Very confident	36 (16.1)	19 (15.1)	13 (16.9)	4 (20.0)	
Confident	123 (55.1)	72 (57.1)	40 (52.0)	11 (55.0)	
Somewhat confident	54 (24.2)	30 (23.8)	19 (24.7)	5 (25.0)	
Not confident	10 (4.5)	5 (4.0)	5 (6.5)	0	
Unable to interpret	0 (0)	0	0	0	
An otherwise healthy 4 year. old patient presents with fevers and headache. His clinical features are not suspicious of bacterial meningitis/encephalitis; however, an LP is done and the CSF bacterial BioFire® results are positive for a bacterial pathogen. Would you start antimicrobials based on positive CSF bacterial BioFire results alone?	-	-	-	-	0.4466
Yes	188 (83.1)	103 (81.5)	68 (87.2)	17 (85.0)	
No	38 (16.8)	25 (19.5)	10 (12.8)	3 (15.0)	
If no, would you start antimicrobials based on positive CSF bacterial BioFire® results in addition to CSF cell count suggestive of meningitis?	-	-	-	-	**0.0043**
Yes	36 (94.7)	25 (100.0)	10 (100.0)	1 (33.3)	
No	2 (5.2)	0	0	2 (66.7)	
In the same patient, would you narrow the antibiotics given the positive bacterial BioFire® test without waiting for culture?	-	-	-	-	0.291
Yes	87 (45.5)	42 (40.4)	36 (51.4)	9 (52.9)	
No	104 (54.4)	62 (59.6)	345 (48.6)	8 (47.1)	
On a 3-week-old febrile infant whose clinical picture is not consistent with bacterial meningitis, would you stop antimicrobials based on positive viral (ex: enterovirus) BioFire® results alone?		-	-	-	0.4185
Yes	82 (36.2)	51 (39.8)	24 (30.8)	7 (35.0)	
No	144 (63.7)	77 (60.2)	54 (69.2)	13 (65.0)	
If no, would you stop antibiotics based on a negative bacterial BioFire® result and normal CSF cell counts without waiting for culture?		-	-	-	0.9182
Yes	29 (20.5)	16 (21.3)	10 (18.9)	3 (23.1)	
No	112 (79.4)	59 (78.7)	43 (81.12)	10 (76.9)	

Abbreviations: LP, lumbar puncture; CSF, cerebrospinal fluid

### Clinical scenarios

Analysis of clinical case scenarios (Table 2) revealed that 188/226 (83%) providers would start antibiotics in a 4-year old with a positive bacterial ME panel result even if clinical features are not consistent with meningitis. This response increased to 95% when a “*CSF cell count suggestive of meningitis”* was provided (Table 2). In this same scenario with “*cell count suggestive of meningitis”*, 45% reported that they would narrow the antibiotics based upon the meningitis/encephalitis panel result without waiting for cultures. In a 3-week old febrile infant whose clinical picture is not consistent with bacterial meningitis, 82/226 (36%) of providers reported that they would stop antibiotics based on the sole presence of positive viral pathogen obtained via the ME panel. Among those who responded they would not stop antibiotics, 29/144 (21%) reported they would stop antibiotics when provided with “*normal CSF cell counts”*.

### Subspecialty analysis

A subset analysis was done between PHM and PEM providers to evaluate for differences between the two groups. This analysis was not performed on PICU providers given the low number of participants from PICU. 48% (61/128) of PHM providers report being aware of the performance characteristic of the ME panel compared to 27% (21/77) of PEM physicians (p = 0.004) (Table 2). There was no difference in reported confidence in interpreting ME panel results between the two groups (p = 0.8). No difference was noted in terms of starting, stopping, or narrowing antibiotics (p = 0.21; p = 0.18; p = 0.15), respectively, between PHM and PEM physicians in the provided clinical scenarios.

## Discussion

This study provides an overview of the ME panel’s current use among pediatric providers in the U.S and highlights the variation in its use and interpretation. 68% of respondents in our survey use the ME panel, but only 25% are aware of ordering guidelines, and 26% have institutional guidelines directing its interpretation. A majority of participants still reported using single organism PCR in addition to the ME panel.

When a new diagnostic test is being introduced, it is imperative that there be clear guidelines on test indications/interpretation, evaluation of the cost-benefit ratio, and an ongoing assessment of the risks and benefits of the test. With the easy availability of the CSF ME panel, overutilization has been noted, with the test being performed in patients with little or no suspicion of CNS infection [13–15]. Furthermore, use of a single organism PCR in addition to the ME panel may add to healthcare costs without providing additional diagnostic information. Although, there may be times where this is necessary, such as HSV PCR testing when this diagnosis is highly suspected, but the ME panel is negative. Test overuse causes unnecessary costs and may also lead to incorrect diagnosis and inappropriate treatment. Though less than half of the providers in our study report being aware of test performance characteristics, the majority reported confidence in interpreting the results. This may further point to overconfidence bias, a known cognitive bias associated with diagnostic inaccuracies and suboptimal management [16]. Increasing providers’ knowledge and providing tools like practice guidelines, diagnostic stewardship, and clinical algorithms are paramount in successfully implementing new testing method. Pairing antimicrobial stewardship with novel diagnostic tools is well-studied in the literature, including blood culture and MEP interpretation. In both instances, introduction of test use guidelines, audit and feedback, and interpretation guidance was associated with decreased antimicrobial use, ancillary testing, and increased antibiotic de-escalation [17, 18]. In the ME panel’s case, these tools ensure maximum clinical benefit (avoid unnecessary antimicrobials, reduce the length of stay, and avoid unnecessary diagnostic tests) without test overuse, inappropriate treatment, and overburdening the health care economy.

In our survey, providers were more likely to start antibiotics in the presence of a positive bacterial ME panel test; however, they hesitated to stop antibiotics with a negative bacterial ME test or with the detection of a viral pathogen. Similarly, less than half of the respondents reported confidence in narrowing the antibiotics based on ME panel results. Potential reasons could include a lack of knowledge and understanding of multiplex PCR by providers and/or concern for false negatives. The ME panel has a sensitivity of 90% and specificity of 98%; however, a small number of false positives and false negatives are reported with the test [19]. These findings suggest that providers’ hesitancy to discontinue antibiotics may in part be due to a lack of knowledge about ME Panel test performance. However, other factors may certainly influence these decisions, including provider experience, pre-test probability, or suspicion for organisms not detected on the ME panel given the clinical scenario. The hesitation in discontinuing antibiotics is in concordance with other studies where physicians start antibiotics more often with a negative viral test but only occasionally stop antibiotics in the presence of viral pathogen [20]. A prior study found that implementation of interpretation guidance for enterovirus testing results was associated with decreased antibiotic use in febrile infants found to have enterovirus meningitis [21]. Similarly, our study results indicate that there is likely an opportunity to provide interpretation guidance and information about ME Panel test performance characteristics to help inform clinical decision making. Inclusion of such tools with diagnostic and antimicrobial stewardship may help achieve desired clinical outcomes. As such, there is a clear role for clinical microbiology laboratory and antimicrobial stewardship teams to work together to develop test use and interpretation guidance to avoid unnecessary antibiotic use. These clinical guidelines or pathways as part of an antimicrobial stewardship team may help provide support to clinicians, especially with patient populations where management can be less straightforward, as is frequently encountered with infants < 2 months old and patients with positive viral targets of unclear significance, such as HHV-6. Studies evaluating such stewardship programs (including use of real-time decision support) indicate potentially positive effects on length of empiric antibiotic and ancillary test utilization [22, 23].

In our study, PHM providers were more aware of the test characteristics compared to PEM providers. This could be attributed to the fact that continuing, narrowing, and stopping antimicrobials falls under the hospitalist realm of practice; thus, they are required to be more knowledgeable. The context in which these two subspecialists encounter a suspected ME patient is also different. Hospitalists have the benefit of observing patients over the clinical course, whereas a PEM provider has the advantage of assessing patients firsthand. Though our study did not find a difference in clinical management between these two subspecialties, further analysis inclusive of subspecialties like ID and PICU is needed to fully elucidate variability. The inclusion of all providers from various specialties involved in the continuum of care is essential while developing clinical tools.

Our study has several limitations. We estimated the number of emails subscribed to the listserves; however, the exact denominator within each listserve changes frequently. Therefore, this might not be an accurate representation of all providers. Our study also has a lower response rate of 4.8% than the average listserve response rate of 8–20% [24–26]. We could not account for potential differences in non-responders to know if our sample represents physicians at large. Furthermore, multiple providers may have responded from a single institution, which may over- or under-represent certain institutional practice. However, we believe this finding highlights intra-institutional practice variation and an opportunity for practice standardization. The high percentage of respondents who reported using the ME panel in our study also raises concern for bias, as people who use the ME panel were more likely to respond to the survey. We only used two specific clinical scenarios on the survey questions with limited information and we did not allow for a free-text response. Thus, our results may not fully reflect what providers would do in real-life clinical scenarios where more clinical and supporting laboratory evidence would be available. The survey was not validated beyond pilot testing with PHM providers, limiting its applicability to PEM and PICU providers. Our clinical vignettes did not specify which bacterial and viral targets were identified, which may have contributed to response variability. Lastly, most of the respondents in our survey were PHM and PEM physicians with limited response from PICU physicians, making the results less generalizable to intensivists. In general, PHM physicians were overrepresented in our sample with limited data from advanced practice providers, which limits generalizability of our study. Furthermore, very few ID physicians responded, and thus our results may not represent practice standards of this subspecialty. However, ID physicians frequently play an important role in clinical decision making in these patients. Despite these limitations, our study provides a good starting point for future research on guideline development for the ME panel and identifies areas for provider education. Comparative analysis of institutions and subspecialty based on the presence or absence of rigorously developed evidence-based guidelines focusing on clinical outcomes and missed diagnosis may provide an answer to this in the future.

## Conclusion

The optimal impact of new diagnostic tools on patient outcomes requires provider education, practice guidelines, and continuous review and feedback. Our study identifies a knowledge gap in these areas for ME panel test. The majority of providers in our study were not aware of guidelines regarding the ME panel test. We did not notice a significant difference between PHM and PEM physicians in clinical management based on ME panel results; however further research is needed to fully evaluate ME panel use variability.

## Electronic supplementary material

Below is the link to the electronic supplementary material.

Supplementary Material 1

## Data Availability

The datasets generated and/or analyzed during the current study are not publicly available. Per a data sharing agreement, deidentified individual participant data cannot be made available. However, data are available from the corresponding author on reasonable request.
