# Supplementary material for: Current state and practice variation in the use of Meningitis/Encephalitis (ME) FilmArray panel in children
Source: BMC Infect Dis. 2022 Oct 31;22:811. doi: 10.1186/s12879-022-07789-2 (PMC9620602; doi:10.1186/s12879-022-07789-2)
Supplement: Supplementary file 1 — Supplementary Material 1 [file 12879_2022_7789_MOESM1_ESM.pdf]

BioFire Testing Pattern Survey

Please complete the survey below.

Thank you!

1.Name of hospital

____

---

2. Characteristics of hospital
(Community hospital= non-teaching hospitals that are not federally funded Combined academic + community = community hospitals with affiliations with universities, colleges, med schools Academic Pediatric hospital within hospital = Children's hospital as a part of bigger system Freestanding children's hospital = geographically, financially, clinically and administratively independent)

◯ Community hospital-University affiliated
◯ Community hospital - Not university hospital
◯ Children's hospital - University affiliated
◯ Children's hospital - Not university affiliated
◯ Others

---

3. Location
(Northeast : CT, ME, MA, NH, RI, NJ, NY, PA, VT Midwest: IL, IN, MI, OH, WI, IA, KS, MN, MI, NE, ND, SD South: DE, FL, GA, MD, NC, SC, VA,DC, WA, KY, AL, MS, TN, AR, LA, OK , TX West : AZ, CO, ID, MT, NV, NM, UT, WY , AK, CA, HI, OR, WA)

◯ US-NE
◯ US-Midwest
◯ US-south
◯ US-West coast
◯ Outside US

---

4. Your current position

◯ Physician (MD/DO)
◯ Advance practice providers

---

5. Your specialty of practice

◯ Hospitalist
◯ Emergency Medicine
◯ Pediatric Intensive Care Unit
◯ Infectious Disease
◯ Neonatal Intensive Care Unit
◯ Others

---

6. How long have you been practicing in this specialty

◯ In-training
◯ 1-2 years
◯ 3-5 years
◯ 6-10 years
◯ 10+ years

7. How long have you been using CSF BioFire (CSF meningitis/encephalitis Filmarray) at your institution?

◯ Not using
◯ 1 or less
◯ 2-3 years
◯ 4 + years

8. Did the CSF BioFire test replace single organism PCR test such as enterovirus or Herpes Simplex Virus (HSV) PCR?

◯ Yes, we no longer use single-organism PCR
◯ No , We still use single-organism PCR in some scenarios
◯ No, we still use single organism PCR in all scenarios

9. Is CSF BioFire test sent out to an external lab?

◯ Yes
◯ No
◯ I don't know

10. What is the average time to result?

◯ < 3 hours
◯ 3-6 hours
◯ 6- 24 hours
◯ >24 hours

11. Are you aware of any institutional guidelines regarding the use (ordering) of CSF BioFire testing?

◯ Yes
◯ No

12. Does your institution provide guidelines for interpreting CSF BioFire results?

◯ Yes
◯ No

13. Are you aware of the performance characteristics (sensitivity/specificity/positive predictive value/negative predictive value) of the CSF BioFire test?

◯ Yes
◯ No
◯ Depends on organism

14. On a scale of 1 to 5, with 5 being VERY confident, how confident are you in interpreting the results of the CSF BioFire test?

◯ 5 -Very Confident
◯ 4-Confident
◯ 3-Somewhat confident
◯ 2-Not confident
◯ 1-Unable to interpret

15. On a 3 week old febrile infant whose clinical picture is not consistent with bacterial meningitis would you stop antimicrobials based on positive viral (ex: enterovirus) BioFire results alone ?

◯ Yes
◯ No

Would you stop antibiotics based on a negative bacterial BioFire result and normal CSF cell counts without waiting for culture?

◯ Yes
◯ No

16. An otherwise healthy 4 yr. old patient presents with fevers and headache. His clinical features are not suspicious of bacterial meningitis/encephalitis, however, an LP is done and the CSF bacterial BioFire results are positive. Would you start antimicrobials based on positive CSF bacterial BioFire results alone?

◯ Yes
◯ No

17. In the same patient (as question no 16) would you start antimicrobials based on positive CSF bacterial BioFire results in addition to CSF cell count suggestive of meningitis?

◯ Yes
◯ No

17. In the same patient (as question no 16) would you narrow the antibiotics given the positive bacterial BioFire test without waiting for culture?

◯ Yes
◯ No

18. Would you like to enter a raffle for a chance to win a $25 gift card?

◯ Yes
◯ No

Please enter your email address

____
